# Label-free method for anti-glucopeptide antibody detection in Multiple Sclerosis

**DOI:** 10.1016/j.mex.2015.03.004

**Published:** 2015-03-12

**Authors:** Feliciana Real-Fernández, Giada Rossi, Francesco Lolli, Anna Maria Papini, Paolo Rovero

**Affiliations:** a Interdepartmental Laboratory of Peptide and Protein Chemistry and Biology–PeptLab, Italy^1^; b Department of Neurosciences, Psychology, Drug Research and Child Health, Section of Pharmaceutical Sciences and Nutraceutics, University of Florence, Via Ugo Schiff 6, I-50019 Sesto Fiorentino, Italy; c Department of Biomedical, Experimental and Clinical Sciences, University of Florence, Viale Morgagni 50, I-50134 Firenze, Italy; d Department of Chemistry “Ugo Schiff”, Via della Lastruccia 13, I-50019 Sesto Fiorentino, Italy

**Keywords:** Antibody detection, Surface plasmon resonance, Serodiagnosis, Label-free immunoassay

## Abstract

Surface plasmon resonance technique is particularly interesting in immunology because it has the potential to visualize label-free antigen-antibody interactions in real-time, thus enabling antibody detection and monitoring. Herein we release the guidelines for the correct use of a method to detect specific antibodies directly in Multiple Sclerosis patients’ sera using a glucopeptide-based label-free biosensor. The protocol describes the strategy employed for the immobilization of glucopeptide antigen onto a gold sensor chip and the evaluation of the specific binding of serum antibodies to the immobilized antigen.

• Label-free method for the real time screening of disease-specific antibodies within a few minutes;
• The described protocol employs small quantities of glucopeptide antigen and blood serum samples saving method-cost;
• Stability of the immobilized glucopeptide antigen guarantees the regeneration of the surface allowing re-use the immunosensor with high automated throughput.

The antibodies detected using the described methodology can be evaluated as biomarkers of Multiple Sclerosis. The SPR detection system is able to characterize antibodies significantly different from those evaluated in the classical enzyme-linked immunosorbent assays (ELISA).

## Graphical abstract

**Figure:**
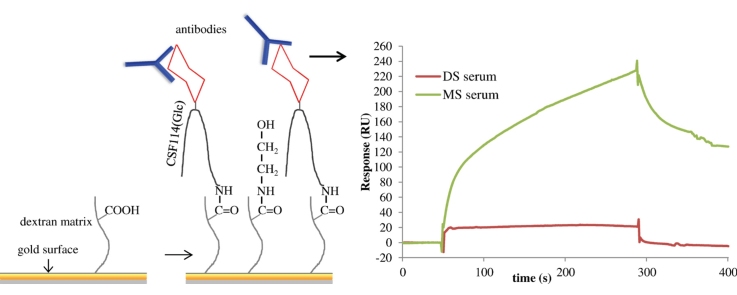

## Method details

One of the major challenges in Multiple Sclerosis diagnosis is the set-up of simple immunodiagnostic methods. In fact, the gold standard for the diagnosis and prognosis of the disease is, up to now, the use of magnetic resonance imaging markers and cerebrospinal fluid analysis. Surface plasmon resonance (SPR) technique has been successfully used to measure the binding of a large number of biomolecular interactions including those of antibodies with cognate antigens [1]. The method for anti-glucopeptide antibody detection in Multiple Sclerosis described herein enables label-free specific antibody detection directly in patients’ sera, using a previously described glucopeptide antigen, termed CSF114(Glc) [2]. A direct comparison of antibody profiles in Multiple Sclerosis patients’ sera by means of enzyme-linked immunosorbent assay (ELISA) and SPR-based biosensor evidenced that, from a diagnostic point of view, results should be independently evaluated [3].

### Glucopeptide antigen immobilization: selection of the immobilization buffer

The glucopeptide CSF114(Glc) was prepared by microwave-assisted solid phase peptide synthesis and further characterized by mass spectrometry and analytical HPLC as described elsewhere [4]. A stock solution of CSF114(Glc) was prepared in pure water (1 μg/μL) and stored at +4 °C. Immediately prior to immobilization procedure, peptide stock solution was diluted in the immobilization buffer to a final concentration of 10 μg/mL.

Sensor chip CM5 (GE Healthcare, Uppsala, Sweden) was inserted into the SPR detector (Biacore T100, GE Healthcare). The running buffer HBS-EP+ 10× (0.1 M HEPES, 1.5 M NaCl, 30 mM EDTA and 0.5% v/v Surfactant P20; yielded pH 7.4 when diluted) was diluted and flowed over the sensor chip channels. All experiments were conducted at +25 °C.

The immobilization buffer was previously selected using the pH scouting protocol, in which the peptide antigen, solved in different buffers, was flowed over the inactive sensor chip for 120 s at a flow rate of 10 μL/min. The regeneration of the chip surface was performed with a pulse of 0.1 M NaOH for 30 s at a flow rate of 10 μL/min after each solution injected. The immobilization buffers were used at pH between 3.5 and the isoelectric point of the antigen in order to achieve the electrostatic pre-concentration of glucopeptide in the dextran matrix of CM5 chip (pre-concentration is favored by low ionic strength in the buffer). The best immobilization buffer was selected injecting the glucopeptide in 10 mM carbonate buffer pH 9.6, PBS buffer pH 7.2, 10 mM, 1 mM and 0.1 mM acetate buffer at pH 4.5, 5.5 and 6.0. Buffers that give irregular sensorgrams or signals with irregular slopes, probably due to ligand aggregation/precipitation or chip saturation, were discarded. The buffer 0.1 mM sodium acetate pH 5.5 presented the highest sensorgram slope and for this reason was selected as the optimal immobilization buffer.

### Glucopeptide antigen immobilization

The flow cell of the sensor chip surface was activated by injecting a 0.4 M 1-ethyl-3-(3-dimethylaminopropyl)-carbodiimide (EDC) and 0.1 M *N*-hydroxysuccinimide (NHS) mixture (50:50), prepared immediately before use, at a flow rate of 10 μL/min during 420 s. The glucopeptide CSF114(Glc) was subsequently injected at 10 μL/min at a concentration of 10 μg/mL in the previously selected immobilization buffer 0.1 mM sodium acetate pH 5.5, using the aim of immobilization procedure to raise a final immobilization level of 800 ± 100 resonance units (RU). Unreacted succinimide groups on sensor chip surface were blocked by injecting 60 s-pulses of 1 M ethanolamine at pH 8.5 at 10 μL/min until complete deactivation. One channel without immobilized ligand was used as reference, to remove the non-specific signal depending on interactions between molecules present in the biological samples and gold on sensor chip surface. At this purpose another different flow cell of the sensor chip was activated and immediately blocked with ethanolamine.

### Monitoring glucopeptide antigen-antibodies interaction: protocol optimization

Human serum samples were thawed till ambient temperature and then diluted 1:100 and/or 1:50 in running buffer. To establish a reproducible method for autoantibody detection, diluted serum samples of a representative high positive patient and a healthy control were injected in triplicate at flow rate of 30 μL/min over the immobilized glucopeptide at different contact times (range 60–240 s). Dissociation was monitored for 60 s by injecting the running buffer suddenly after samples at a flow rate of 30 μL/min. Interactions were recorded as separate sensorgrams and measurements registered 15 s after the end of each sample injection. Responses were measured in resonance units (RU) as the difference between reference and active channel.

The selected optimal conditions include the 1:50 sera dilution injected for 240 s, which presented increased signal differences between positive and negative samples maintaining low non-specific interactions in the reference channel. After each sample injection, surface was regenerated with two 60 s pulses of a solution 100 mM NaOH allowing the complete removal of specifically and non-specifically bounded biological material from the surface. Following this protocol all further experiments were performed not over and above 100 measurements per channel.

The analytical variability of the assay was checked screening one characteristic high positive serum at dilution 1:50 testing successively the same sample (15 runs) or in different experiments (12 runs performed once a week). The within-assay and between-assay coefficients of variation (SE/mean) were below 5% and then considered as a low signal average. This positive sample, and another sample selected as negative control, can be used as control each 15 samples measurement during the screening of a pool of sera, verifying the stability of the probe upon a large number of cycles. For each measurement a sample volume of 150 μL was employed, thus small amount of 3 μL of patient serum was required for each assay.

### Calibration curve with purified antibodies

**Fig. 1:**
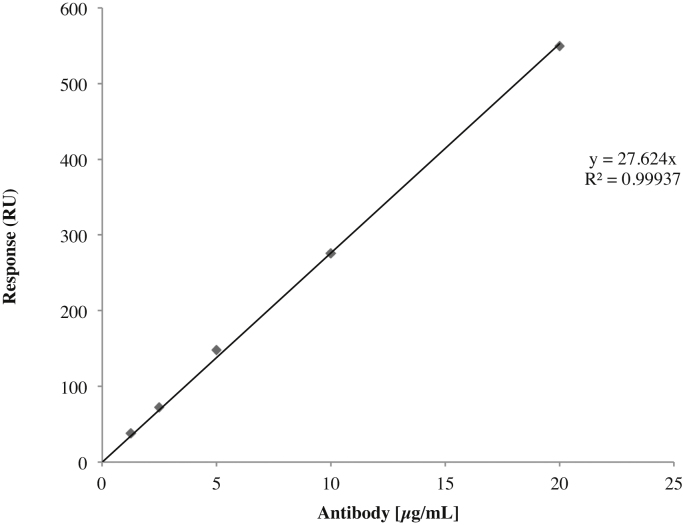
Measured values registered at the different concentrations of purified anti-CSF114(Glc) antibodies.

Immunoaffinity purified anti-CSF114(Glc) antibodies [5] from one high positive control serum were injected at five different concentrations (from 1.25 μg/mL to 20 μg/mL) on the flow channels. The running buffer was then flushed for 60 s and chip surface was regenerated with two injections of 100 mM NaOH during 60 s each one. All the steps were performed at a flow rate of 30 μL/min. The resulting resonance signal was plotted against antibodies concentration to give the calibration curve reported in Fig. 1. The obtained results showed a direct correspondence between the instrumental signal and the antibodies concentration inside the sample, indicating that the herein proposed SPR based method can be successfully applied to detect and to quantify polyclonal antibodies in human serum specifically interacting with the immobilized glucopeptide antigen.

### SPR binding studies with human sera

The specific antibody reactivity of Multiple Sclerosis patients’ sera against the immobilized CSF114(Glc) antigen was evaluated according to the above described optimized protocol. Each sample was tested in triplicate at a flow rate of 30 μL/min. Human sera were diluted 1:50 in running buffer and were injected during 240 s followed by 60 s of buffer flow. After each measurement, chip surface was regenerated injecting two pulses of a 100 mM NaOH solution during 60 s. Interaction of samples with sensor chip flow cells were monitored as separate sensorgrams and measurements were taken 15 s after the end of each injection. The anti glucosylated peptide responses, measured in RU and resulting from the signal difference between active and reference channel, allowed a significant quantification of specific antibodies present in patients' sera as compared to normal blood donors.

SPR-based analyte quantification is hard to perform when injecting a complex biological matrix as sample. The present experiments demonstrated that, thanks to an appropriate covalent immobilization strategy and to carefully optimized run parameters, SPR-based immunoassays can be successfully used to specifically detect antibodies of proven disease relevance, directly in patients’ sera without the need of any previous purification step.
